# Aneuploidy Underlies Tolerance and Cross-Tolerance to Drugs in Candida parapsilosis

**DOI:** 10.1128/Spectrum.00508-21

**Published:** 2021-10-06

**Authors:** Feng Yang, Hui Lu, Hao Wu, Ting Fang, Judith Berman, Yuan-ying Jiang

**Affiliations:** a Department of Pharmacy, Shanghai Tenth People’s Hospital, School of Medicine, Tongji University, Shanghai, China; b Shmunis School of Biomedical and Cancer Research, The George S. Wise Faculty of Life Sciences, Tel Aviv University, Tel Aviv, Israel; The Ohio State University

**Keywords:** aneuploidy, aureobasidin A, *Candida parapsilosis*, cross-adaptation, ER stress, tunicamycin, stress adaptation

## Abstract

*Candida* species are the most common human fungal pathogens worldwide. Although C. albicans remains the predominant cause of candidiasis, infections caused by non-albicans *Candida* species, including C. parapsilosis, are increasing. In C. albicans, genome plasticity has been shown to be a prevalent strategy of adaptation to stresses. However, the role of aneuploidy in C. parapsilosis is largely unknown. In this study, we found that six different aneuploid karyotypes conferred adaptation to the endoplasmic reticulum stress inducer tunicamycin (TUN) in C. parapsilosis. Interestingly, a specific aneuploidy including trisomy of chromosome 6 (Chr6x3) also enabled cross-tolerance to aureobasidin A (AbA), a sphingolipid biosynthesis inhibitor. Consistent with this, selection on AbA identified adaptors with three different aneuploid karyotypes, including Chr6x3, which also enabled cross-tolerance to both AbA and TUN. Therefore, as in other *Candida* species, recurrent aneuploid karyotypes enable the adaptation of C. parapsilosis to specific stresses, and specific aneuploidies enable cross-adaptation to different stresses.

**IMPORTANCE** Candida parapsilosis is an emerging human fungal pathogen, especially prevalent in neonates. Aneuploidy, having uneven numbers of chromosomes, is a well-known mechanism for adapting to stress in Candida albicans, the most common human fungal pathogen. In this study, we exposed C. parapsilosis to two very different drugs and selected for rare cells that grew in one of the drugs. We found that the majority of isolates that grew in the drugs had acquired an extra copy of one of several aneuploid chromosomes and that specific aneuploid chromosomes appeared in several independent cell clones. Importantly, an extra copy of chromosome 6 was detected following selection in either one of the drugs, and this extra chromosome conferred the ability to grow in both drugs, a property called cross-adaptation, or cross-tolerance. Thus, this study highlights the genome plasticity of C. parapsilosis and the ability of an extra copy of a single chromosome to promote cell growth in the presence of more than one drug.

## INTRODUCTION

*Candida* species are commensal colonizers of human skin, the oral cavity, and the gastrointestinal tract and also are the most common cause of human fungal infections, particularly among immunosuppressed and immunocompromised individuals (1). *Candida* species are the fourth leading cause of nosocomial bloodstream infections in the United States (2), and invasive infections have a high mortality of up to 70% (3). In most cases, C. albicans is the leading cause of candidiasis, although in recent years, the incidence of opportunistic infections caused by non-albicans *Candida* species is also rising. In many countries, including the United States, Europe, Canada, and Latin America, C. parapsilosis is the second most commonly isolated *Candida* species from blood samples (reviewed in reference 4). C. parapsilosis also is notorious as one of the leading causes of catheter-related infections (3, 5) and ranks second or third as the most commonly isolated *Candida* species in intensive care units, especially in neonatal intensive care units (6–9).

Aneuploidy, the state of unbalanced chromosome copy number, is a common mechanism of adaptation to stresses in C. albicans (reviewed in references 10–12). The first classic example is utilization of the l-sorbose, a toxic sugar, as the sole carbon source via chromosome 5 (Chr5) monosomy (Chr5x1) (13). Another classic example is the acquisition of an isochromosome consisting of two left arms of Chr5 in fluconazole-resistant clinical isolates (14). Trisomy of Chr4 also causes fluconazole resistance in one clinical isolate (15). Aneuploidy is the major mechanism of adaptation to the first-line antifungal drug caspofungin in C. albicans (16, 17). *In vivo* passage in mice via the bloodstream, oral cavity, or gastrointestinal tract induces genome instability and selects for particular aneuploid karyotypes (18–21). Of note, during genetic manipulation, aneuploidy can occur, especially in response to heat shock, yielding unexpected karyotypes and associated phenotypes in laboratory strains (22–26). Importantly, aneuploidy not only causes tolerance to particular stresses, but it also causes cross-adaptation to unrelated stresses. For example, both l-sorbose and caspofungin select for Chr5x1; therefore, Chr5x1 causes cross-tolerance to l-sorbose and caspofungin (16, 27). Exposure to caspofungin or to the chemotherapeutic drug hydroxyurea selects for Chr2 trisomy (Chr2x3), and the ability to grow on both drugs is lost when the trisomy is lost. Thus, Chr2x3 promotes cross-tolerance to caspofungin and hydroxyurea (17). Recently, we found that C. albicans can tolerate trisomy of each chromosome, highlighting its genome plasticity (28).

Aneuploidy is also found in non-albicans *Candida* species. In C. glabrata, sequential bloodstream isolates exhibit karyotype variations and alterations of azole susceptibility (29). Gene copy number variation and chromosome size variations due to reciprocal chromosomal translocations and recombination within tandem arrays of repeated genes are also found in clinical isolates (30). The presence of a new minichromosome in some clinical isolates is associated with fluconazole resistance (31). In C. tropicalis, polyploidy and single-chromosome aneuploidy (trisomy of scaffold 8 and scaffold 4) were found in two clinical and two lab isolates (32). In C. auris, clinical isolates from the four major clades have diverse karyotypes and can undergo rapid karyotype and nuclear DNA content alterations during stress exposure (33). For example, daily passaging in fluconazole recurrently selected for resistant progeny with ChrV disomy (34). Thus, aneuploidy is a common stress response mechanism in several *Candida* species.

C. parapsilosis, like C. albicans and C. tropicalis, is diploid, and a reference sequence for strain CDC317 is available (35). Based on propidium iodide staining and analysis of amplified sequences in 13 natural C. parapsilosis isolates, Fundyga et al. concluded that C. parapsilosis isolates have highly diverse nuclear DNA content, with genome sizes ranging from half to the same size as related diploid yeast genomes. Thus, haploidy and aneuploidy may be common in clinical C. parapsilosis isolates (36). However, no direct evidence of stress-induced aneuploidy has been reported in C. parapsilosis.

Tunicamycin (TUN) is an antibiotic made by Streptomyces clavuligerus and Streptomyces lysosuperficus. It blocks N-linked glycosylation by inhibiting the transfer of UDP-*N*-acetylglucosamine to dolichol phosphate in the endoplasmic reticulum (ER) of eukaryotic cells, resulting in misfolded proteins and ER stress (37). In the model organism Saccharomyces cerevisiae, selection on TUN caused aneuploidy of several chromosomes, with recurrent appearance of chromosome 2 disomy (38).

Sphingolipids are emerging as a potential new target for the development of antifungal agents (39). Aureobasidin A (AbA), a natural compound that inhibits fungus-specific inositol phosphorylceramide (IPC) synthase, has potent antifungal activity against several *Candida* species, including C. parapsilosis (40). However, the mechanism of C. parapsilosis adaptation to AbA is not known.

In this study, we performed direct selection of C. parapsilosis drug “adaptors,” cells that acquired the ability to grow better than their parents in inhibitory concentrations of two unrelated drugs—tunicamycin (TUN), an inducer of endoplasmic reticulum (ER) stress, and aureobasidin A (AbA), an inhibitor of sphingolipid synthesis. Most of these adaptors were aneuploid, based on mapping of their whole-genome sequences. This yielded several karyotypes, some of which suggest potential translocations between chromosomes 1 and 2 and between chromosomes 3 and 8 in the parent strain used here, relative to the C. parapsilosis reference sequence. Chr6 trisomy was recurrently detected in isolates selected from exposure to either TUN or AbA, and it conferred the ability to grow on both drugs together. This suggests that Chr6 trisomy provides cross-tolerance to both TUN and AbA and suggests that aneuploidy is a common mechanism of stress adaptation in C. parapsilosis.

## RESULTS

### Obtaining tunicamycin adaptors.

**FIG 1:**
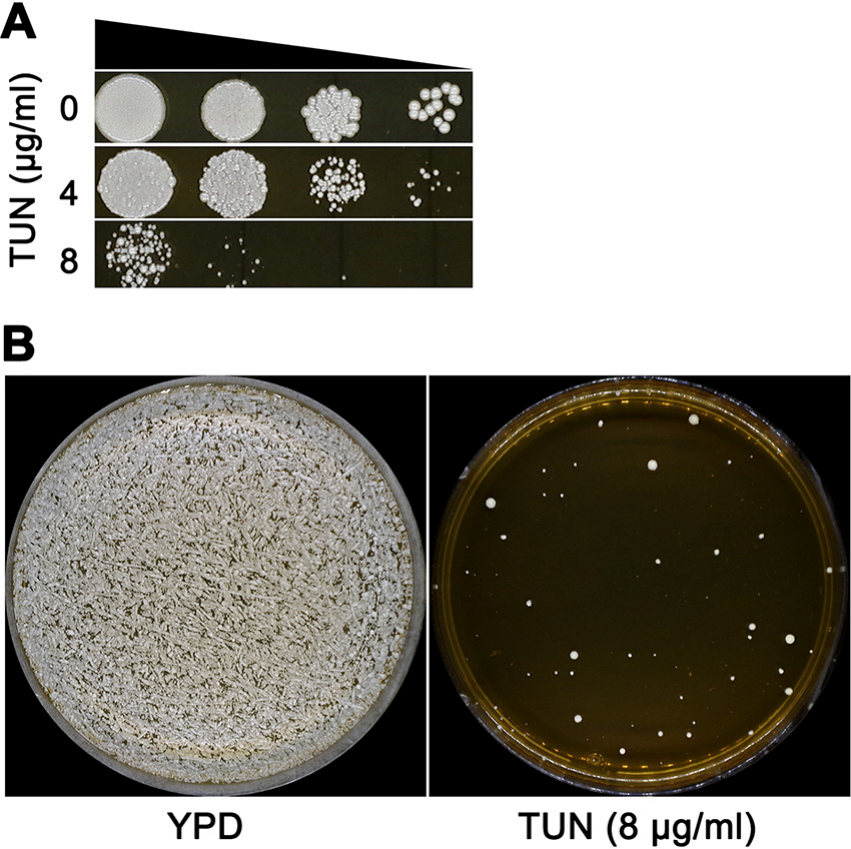
Selecting for tunicamycin adaptors. (A) Susceptibility of parent YJB-T12108 to tunicamycin (TUN) was measured by spot assay on YPD plates supplemented with the TUN concentrations indicated. (B) Approximately 1 × 10^5^ cells of parent YJB-T12108 were spread on YPD (control) or YPD plates supplemented with 8 μg/ml TUN. The plates were incubated at 37°C for 2 days (panel A) or 3 days (panel B) and then photographed.

Susceptibility of strain YJB-T12108 to TUN was measured using spot assays, with growth obviously inhibited at 8 μg/ml of TUN. Growth was reduced mildly at 4 μg/ml of TUN (Fig. 1A). Thus, we screened for TUN adaptors by plating approximately 1 × 10^5^ cells on yeast extract-peptone-dextrose (YPD) agar plates supplemented with 8 μg/ml of TUN. After 3 days of growth at 37°C, approximately 38 colonies (approximate frequency, 3.8 × 10^−4^) were detected on the plate (Fig. 1B), and 18 of them (T1 to T18) were randomly chosen for whole-genome sequencing.

### TUN adaptors are aneuploid and tolerant.

**FIG 2:**
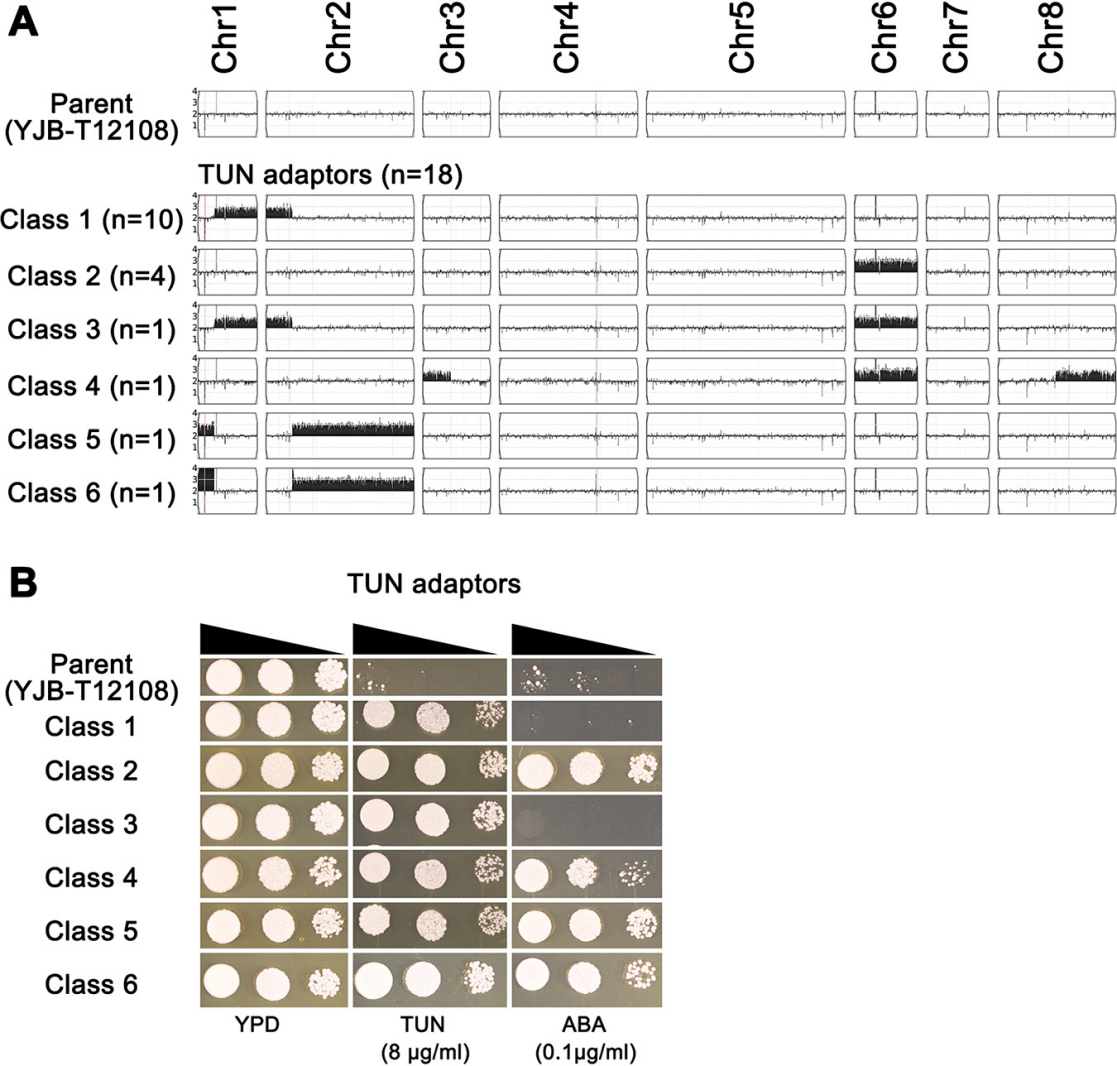
Karyotype classes and tolerance profiles of TUN adaptors. (A) Karyotypes representing the classes of TUN adaptors with the number of adaptors with the same karyotype indicated. The karyotypes were visualized using YMAP (41). The *y* axis indicates the chromosome copy number (log_2_ ratio of adaptor/diploid parent). The *x* axis indicates the positions of the reads on each chromosome for reference strain CDC317. The karyotype of the parent YJB-T12108 is also shown. (B) Adaptors representing each unique karyotype were spot assayed for tolerance to TUN and AbA; 3 μl of 10-fold serial dilutions was spotted on YPD agar plates supplemented with drugs at the concentrations indicated in the figure, and plates were incubated at 37°C for 2 days before photographing.

Alignment of the DNA sequences onto the C. parapsilosis reference genome sequence using YMAP (41) revealed a diversity of aneuploid karyotypes (see Fig. S1 in the supplemental material and Fig. 2A), which were classified into six classes. Four of these classes of adaptors (class 1 [10 isolates], class 3 [1 isolate], class 5 [1 isolate], and class 6 [1 isolate]) had amplified fragments of both Chr1 and Chr2 in a pattern that appears to be inverted relative to one another. The majority (class 1 and class 3) were trisomic for ∼0.64 Mb from the right end of Chr1 (Chr1R), together with ∼0.40 Mb of the left end of Chr2 (Chr2L) to yield Chr1R/2Lx3. The two inverted examples (class 5 [T7] and class 6 [T14]) amplified ∼1.8 Mb from the right end of Chr2 (Chr2R) along with ∼0.25 Mb from the left end of Chr1 (Chr1L), with class 5 having one extra copy (Chr1L/Chr2Rx3) and class 6 being tetrasomic for Chr1L. The junctions between aneuploid and euploid copy number in all four of these classes appear to be the same, suggesting that the parent strain carried a reciprocal translocation between Chr1 and Chr2 similar to the translocations observed in C. glabrata (30), with one of the two Chr1/Chr2 translocation partners amplified in each case. However, this does not fully explain the tetrasomy of the Chr1Lx4 fragment in class 6.

Class 2 adaptors (T8, T12, T17, and T18) were trisomic for full-length Chr6 (Chr6x3). One class 3 adaptor (T3) had both the Chr1R/2Lx3 karyotype changes of class 1 and the Chr6x3 of class 2. Class 4 adaptors also had Chr6x3, which also had duplicated ∼0.43 Mb from the left end of Chr3 and ∼0.92 Mb from the right end of Chr8 (Chr3L/8Rx3). Thus, the majority of adaptors (classes 1 and 3, *n* = 11) carried Chr1R/2Lx3, and the reciprocal Chr1L/2Rx3 was only seen in two classes (*n* = 2, classes 5 and 6). The second most common set of adaptors included Chr6x3 aneuploidy (classes 2, 3, and 4; *n* = 6), either alone (class 2, *n* = 4) or in combination with Chr1R/2Lx3 or with Chr3L/8R (classes 3 and 4) (which was not seen alone). The least frequent were Chr1L/2Rx3 (*n* = 1) and Chr3L/8R with Chr6x3. In summary, all six classes of aneuploid isolates involved trisomy of two Chr1/2 reciprocal fragments, or Chr6x3, either alone or in combination with Chr1R/2Rx3 or Chr3L/8Rx3.

Spot assays were performed to compare the ability of the adaptors and the parent strain to grow in the presence of different drugs (TUN and AbA). All the TUN adaptors grew better than the parent on YPD + TUN plates. In addition, seven TUN adaptors (T5, T7, T8, T12, T14, T17, and T18), belonging to four classes of karyotypes (class 2 [Chr6x3], class 4 [Chr6x3 + Chr3L/8Rx3], class 5 [Chr1L/2Rx3], and class 6 [Chr1Lx4/Chr2Rx3]) were cross-tolerant to AbA (Fig. S2 and Fig. 2B). Interestingly, the most frequent adaptors, class1 (Chr1R/2Lx3) and class 3 (Chr1R/2Lx3 + Chr6x3), were not cross-tolerant. These results are consistent with the idea that Chr6x3 may provide cross-adaptation to AbA but that Chr1L/2Rx3 antagonizes this AbA adaptation. Nonetheless, TUN adaptors acquired trisomy of Chr1, Chr2, and Chr6, and a subset of these isolates was associated with cross-adaptation to AbA as well.

### AbA adaptors are mostly aneuploid and tolerant.

**FIG 3:**
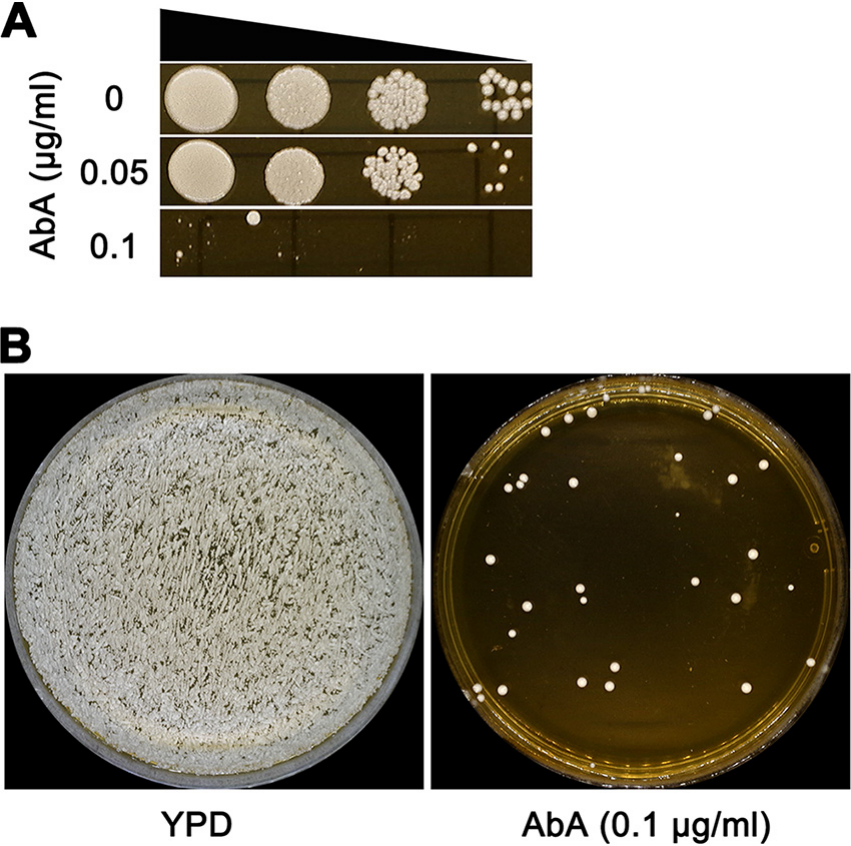
Selecting for aureobasidin A adaptors. (A) Susceptibility of parent YJB-T12108 to aureobasidin A (AbA) was measured by spot assay on YPD plates supplemented with AbA. (B) Approximately 1 × 10^5^ cells were spread on YPD (control) or a YPD plate supplemented with 0.1 μg/ml AbA. The plates were incubated at 37°C for 2 days (panel A) or 3 days (panel B) and then photographed.

To ask if selection on AbA yielded classes of aneuploidy previously seen for TUN adaptors, we first determined the susceptibility of parent strain YJB-T12108 to AbA by spot assay and determined that 0.1 μg/ml AbA (Fig. 3A) inhibited growth substantially. Thus, we selected for adaptors by spreading ∼1 × 10^5^ cells onto YPD + AbA (0.1 μg/ml) agar and incubating the plates for 3 days at 37°C. Approximately 33 colonies (frequency, 3.3 × 10^−4^) were obviously visible on the plate (Fig. 3B), and 18 of them (A1 to A18) were randomly chosen for further analysis. In spot assays, 15 adaptors were more tolerant than the parent to AbA, and the remaining three adaptors (A2, A15, and A17) were not tolerant. All 15 tolerant adaptors were sequenced (Fig. S3B).

**FIG 4:**
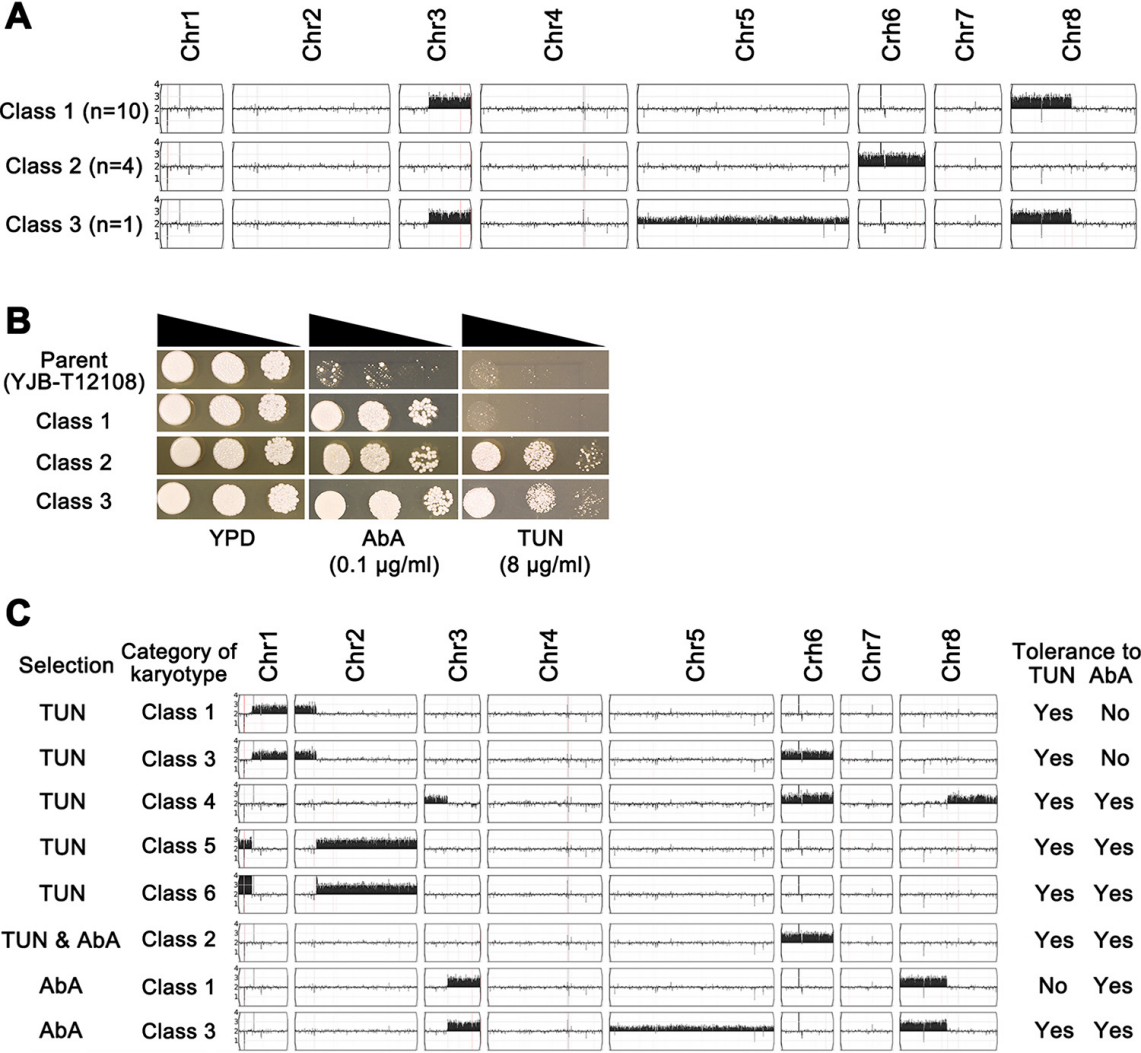
Karyotype classes and tolerance profiles of AbA adaptors. (A) Among the 15 tolerant AbA adaptors were 3 classes of karyotypes. The number of adaptors with the same karyotype is indicated. (B) Representatives of the three classes of karyotypes were spot assayed for tolerance to AbA and TUN at the concentrations indicated in the figure, and plates were incubated at 37°C for 2 days before photographing. (C) Summary of all karyotypes found among tunicamycin (TUN) and aureobasidin A (AbA) adaptors (middle panel); the selection regime in which they appeared (first column in the left panel), classification of karyotypes (second column in the left panel), and tolerance profiles to TUN and AbA (right two columns) are shown.

The adaptors were categorized into three classes (Fig. 4A and B), based on the karyotypes and tolerance to drugs. Interestingly, the most frequent adaptors (class 1 [A1, A3, A4, A5, A6, A7, A9, A11, A14, and A16]) were tolerant to AbA and not tolerant to TUN. Class 1 adaptors had duplicated fragments of both Chr3R and Chr8L. The breakpoint junctions were immediately adjacent to the breakpoints in Chr3L and Chr8R seen in TUN adaptor class 4. Importantly, these class 4 adaptors also included Chr6x3 and were cross-tolerant to AbA and TUN. Four adaptors (A10, A12, A13, and A18) had Chr6x3 and were cross-tolerant to AbA and TUN. One adaptor (A8, class 3) included the Chr3R/8Lx3 from class 1 in addition to trisomy of Chr5 (Chr5x3) and, unlike class 1 adaptors, it was tolerant to AbA and cross-tolerant to TUN (Fig. 4B and Fig. S3A). Thus, the predominant class 1 karyotype did not confer cross-tolerance, while class 2 (Chr6x3) and class 3 (Chr3R/Chr8Lx3 + Chr5x3) enabled cross-tolerance to TUN. Given the recurrent appearance of Chr3/8 fragments among the TUN and AbA selected colonies, we suggest that these two chromosomes also underwent a reciprocal translocation in parent strainYJB-T12108 relative to the C. parapsilosis reference genome, and these two translocated chromosomes appear to provide tolerance to either TUN (for Chr1R/2Lx3) or AbA (for Chr3R/8Lx3) but not cross-tolerance, while Chr6x3 alone appears to provide cross-tolerance to both TUN and AbA in C. parapsilosis.

### Instability of tolerance.

**FIG 5:**
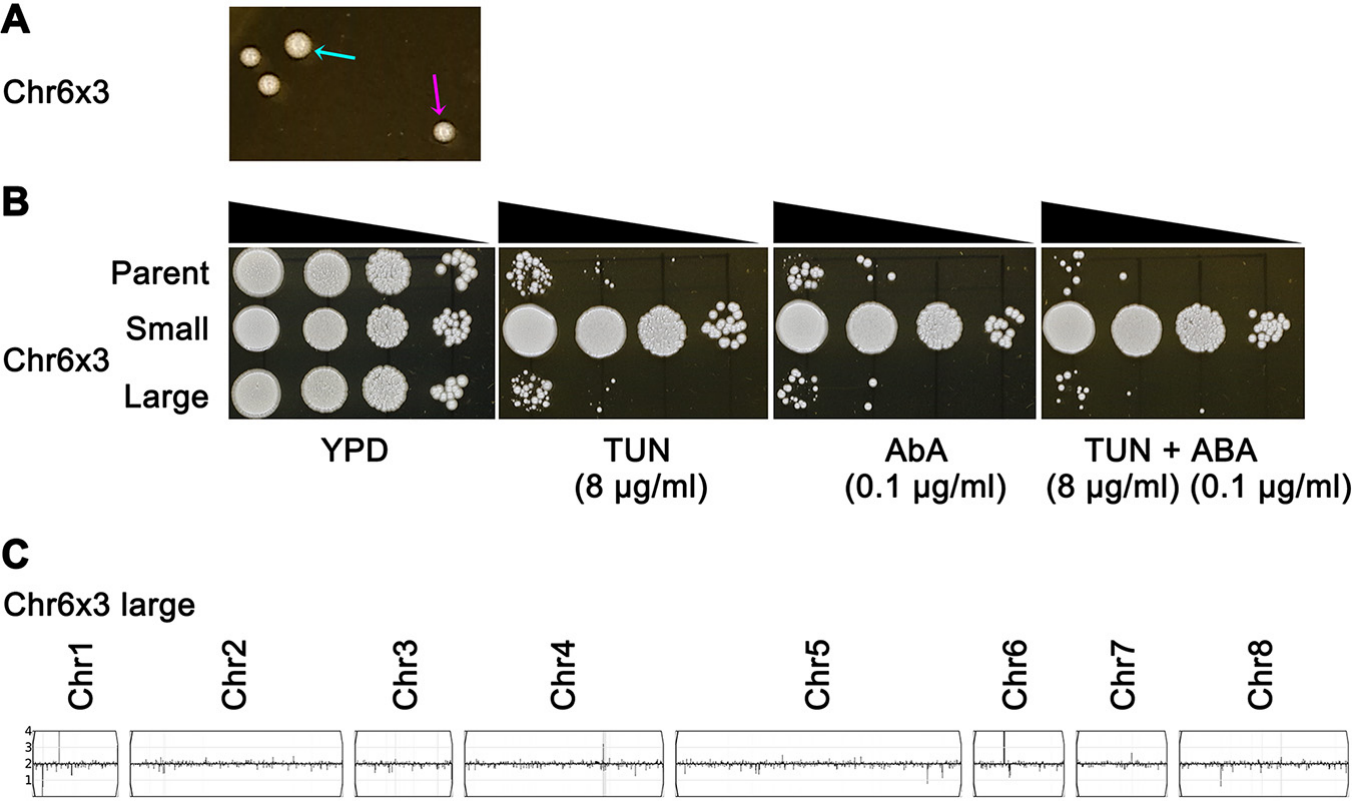
Drug tolerance in the aneuploid adaptor is unstable. Cells of one Chr6x3 adaptor were spread on YPD plates directly from freezer stocks. The plate was incubated at 37°C for 48 h. (A) The magenta arrow indicates the small colony. The cyan arrow indicates the large colony. A spot assay was performed to compare small and large colonies as well as the parent, YJB-T12108, for tolerance to TUN, AbA, and both drugs together (TUN+AbA) at the concentrations indicated. (B) The plates were incubated at 37°C for 48 h and then photographed. (C) The genome sequence karyotype of the large colony was visualized using YMAP.

C. albicans aneuploids exhibit instability by reverting to the euploid state (17, 27, 28), with euploid revertants forming larger colonies on YPD (no drug) plates than the parental aneuploids, presumably because aneuploids are slightly less fit in the absence of drug selection. To ask if aneuploid C. parapsilosis adaptors also revert to the euploid state, we spread approximately 1,000 cells of a Chr6x3 adaptor onto several YPD plates and observed colony sizes after 48 h of growth at 37°C. Most colonies were small, like their aneuploid parent (Fig. 5A, magenta arrow), and a few (∼0.3%) larger colonies also appeared (Fig. 5A, cyan arrow). One small colony and one large colony were selected randomly and tested for tolerance to TUN and AbA. Spot assays indicated that the small colony was tolerant to TUN and AbA, and the large colony had lost the tolerance phenotype (Fig. 5B). Furthermore, in broth microdilution assays, the parent strain YJB-T12108 and the large colony had the same TUN (8 μg/ml) and AbA (0.1 μg/ml) MICs, while the small colony was more tolerant, with TUN and AbA MICs 2-fold higher ([Fig. S4]). This suggests that the large colonies had reverted to the state for both colony size and MIC. Sequencing the genome of the large colony revealed that it was euploid (Fig. 5C). Thus, as with C. albicans, aneuploidy, here trisomy of Chr6, can confer drug tolerance. Furthermore, the aneuploid state is partially unstable (with loss rates of 0.03% to ∼0.4% in the absence of drug selection for 2 days).

In addition to Chr6x3, other aneuploids also yielded small (Fig. S5A, magenta arrows) and large colonies (Fig. S5A, cyan arrows) on YPD. Only the small colonies were drug tolerant, while the large colonies were not (Fig. S5B). Genomic DNA content of the small and large colonies was measured by flow cytometry with propidium iodide (42). Cells from the small colonies all had >2N genomic DNA content, and all the large colonies had genomic DNA content of ∼2N, similar to the parent strain (Fig. S5C). This indicates that the small colonies carried one or more extra chromosomes, and the large colonies had lost the extra chromosomes.

## DISCUSSION

Here, we found that aneuploidy confers tolerance to two different inhibitors, TUN, which induces ER stress, and AbA, which inhibits sphingolipid biosynthesis. Most adaptors to TUN or AbA acquired recurrent trisomy of fragments from two chromosomes (Chr1/2 or Chr3/8, respectively). The fact that the reciprocal fragments were also seen and that the breakpoint junctions (where copy number went from 3 to 2) were immediately adjacent to each other, suggests that at least two reciprocal translocations occurred in the parent strain used for these studies relative to the reference strain used for mapping the sequencing data. We also found that Chr6x3 conferred tolerance to both TUN and AbA, as well as to both drugs together (Fig. 5B) and that some other aneuploid combinations also conferred cross-tolerance to both drugs. However, the most frequently isolated adaptor classes (class 1 of TUN adaptors and class 1 of AbA adaptors) were not cross-tolerant (Fig. 4C). This is consistent with the idea that different genes on the amplified chromosomes are important for tolerance to each drug. However, we cannot rule out the possibility that some genes on Chr6, when trisomic, may confer tolerance to both drugs simultaneously. Further work will be required to address this question directly.

TUN inhibits N-linked glycosylation of proteins, protein folding, and transit through the ER and is often used as a prototypic ER stress inducer (36, 43). The ER is the major compartment of correct folding and posttranslational modification of proteins destined to other organelles, the plasma membrane and the extracellular compartment. If the ER protein folding capacity becomes overwhelmed, ER stress ensues and is characterized by misfolded proteins that accumulate inside the ER lumen (36). Resistance to TUN in S. cerevisiae is often conferred by specific aneuploid chromosomes (38); four of five TUN-resistant mutants were aneuploid, each having a different whole-chromosome aneuploidy pattern involving at least two chromosomes (38). Recently, we found that adaptation to TUN also emerges via aneuploidy in C. albicans; 93.5% (58 out of 62) adaptors in this study carried Chr2 trisomy, and only 5 of these had another aneuploidy in addition to Chr2 trisomy (44). Thus, aneuploidy appears to be a common adaptive genome change that can confer tolerance or resistance to tunicamycin. It is also possible that TUN inhibition of ER processes may stimulate chromosome mis-segregation events that yield aneuploid progeny, some of which (depending on the constellation of chromosomes that they inherit) are better able to grow on tunicamycin.

AbA is a cyclic depsipeptide antifungal that targets inositol phosphorylceramide (IPC) synthase, a process essential in fungi but not required in mammalian cells (reviewed in reference 39). In S. cerevisiae, IPC synthase is encoded by *AUR1*, And *AUR1* mutation causes tolerance to AbA (45). In C. albicans, deletion of one allele of AUR1 causes hypersensitivity of AbA (46). Similarly, in Aspergillus, mutation of the *aurA* gene, which is homologous to yeast *AUR1*, causes high levels of AbA tolerance (47). In addition to *AUR1*, *PDR16* also is associated with AbA tolerance. Overexpression of *PDR16*, which encodes a phosphatidylinositol transfer protein (PITP), causes high levels of AbA tolerance in S. cerevisiae (48). Here, we first observed C. parapsilosis Aba tolerance associated with Chr6x3 that emerged during selection for TUN tolerance. Upon direct selection with an inhibitory concentration of AbA, most (15 of 18) adaptors were AbA tolerant, and all of these were aneuploid, with three different karyotypes. Thus, aneuploidy is clearly a frequent and mechanism of adaptation to AbA as well as to TUN.

Aneuploidy usually confers adaptation to specific stresses by affecting the copy number of a specific gene(s) on the aneuploid chromosome (17, 49–52). In S. cerevisiae, three genes (*ALG7*, *PRE7*, and *YBR085C-A*) are associated with tolerance to TUN (38). In the genome of the reference C. parapsilosis strain, CDC317, the orthologs of *ScALG7* and *ScPRE7* are *CpALG7* (*CPAR2_104220*) and *CpPRE7* (*CPAR2_403290*), respectively, while an ortholog of *YBR085C-*A is not evident in the CDC317 genome. *CpALG7* maps within the trisomic Chr2R fragment in TUN class 5 and class 6 adaptors. Future studies will address the question of whether an extra copy of this gene is sufficient to explain the elevated tolerance of the class 5 and class 6 isolates. Nonetheless, the other 16 adaptors did not have extra copies of C*pALG7* or *CpPRE8*. Thus, TUN tolerance in most adaptors likely occurs via alternative or indirect mechanisms that remain to be elucidated.

In S. cerevisiae, two genes, *AUR1* and *PDR16*, are associated with tolerance to AbA (45, 48); the C. parapsilosis *CDC317* orthologs are *CpAUR1* (*CPAR2_302580*) and *CpPDR16* (*CPAR2_105110*), respectively. *CpAUR1* is in the trisomic region of Chr3R in 11 of the AbA adaptors (part of Chr3R/8Lx3), and it will be important to test the hypothesis that an extra copy of *CpAUR1* is sufficient to confer AbA tolerance, given that overexpression of *AUR1* confers tolerance to AbA in S. cerevisiae (53). Importantly, most of the adaptors did not have extra copies of C*pALG7* or *CpPRE8*. The cross-tolerance to TUN of these aneuploid adaptors likely occurs via alternative or indirect mechanisms that remain to be elucidated.

Cross-adaptation is likely due to different genes that confer benefits under each of the inhibitory conditions. *CpPDR16* maps to the region of Chr2R that is trisomic in TUN class 5 and class 6 adaptors that also are tolerant to AbA. Thus, it is tempting to speculate that the cross-tolerance to both TUN and AbA of class 5 and class 6 adaptors is due to extra copies of both *CpALG7* and *CpPDR16* on the Chr2R trisomy. Intriguingly, four of the AbA adaptors and four of the TUN adaptors had Chr6 trisomy and were cross-tolerant to AbA and TUN, yet none of the known genes associated with TUN or AbA responses (*CpALG7*, *CpPRE7*, *CpAUR*1, and *CpPDR1 6*) map to Chr6. Thus, here too, mechanisms of tolerance and cross-adaptation remain to be discovered. In addition, when Chr6x3 was coupled with Chr1R/2Lx3 in the class 3 TUN adaptor, there was no cross-adaptation to AbA. This suggests that either Chr1R/2Lx3 carries a gene that antagonizes the Chr6x3 mechanism of AbA tolerance and/or the cross-adaptation mechanism conferred by Chr6x3 is highly sensitive to the stoichiometry of gene products encoded on Chr1L/2R.

In addition to trisomy of Chr6, other aneuploidies (e.g., Chr3R/8Lx3+Chr5x3) also confer cross-tolerance, indicating that both TUN and AbA selection can appear with acquisition of a single aneuploidy. This resembles the situation in C. albicans, where Chr2 trisomy confers cross-adaptation to both caspofungin, an antifungal drug, and to hydroxyurea, an inhibitor of ribonucleotide reductase used in cancer chemotherapies (17), as well as to TUN (44). Where it has been tested, different genes on the same chromosome confer adaptation to the different drugs (17, 27, 54). However, some genes may influence responses to two of the drugs; for example, in C. albicans, *MKK2* is required for tolerance to both TUN and caspofungin, but not for tolerance to hydroxyurea (44).

Taken together, the results of this study revealed several diverse C. parapsilosis karyotypes that confer tolerance and, in some cases, cross-tolerance to two unrelated inhibitors, TUN and AbA. The different phenotypes largely associated with the different recurrent karyotypes, consistent with the idea that aneuploidy drives many of the tolerance responses. We suggest that aneuploidy is a common mechanism of adaptation and cross-adaptation to stresses in C. parapsilosis, as it is in other *Candida* species.

## MATERIALS AND METHODS

### Strains and growth conditions.

C. parapsilosis clinical isolate YJB-T12108 was used as the parent to obtain drug adaptors. Stock cultures of all strains were preserved in 35% glycerol and maintained at −80°C. Unless otherwise specified, cells were routinely grown on yeast extract-peptone-dextrose (YPD) agar medium (1% [wt/vol] yeast extract, 2% [wt/vol] peptone, 2% [wt/vol] d-glucose, and 2%[wt/vol] agar) at 37°C.

### Spot assay.

As described previously (28), strains were streaked onto YPD agar and incubated at 37°C for 36 h, and several colonies were chosen randomly and suspended in distilled water. Cell densities were measured using a hemocytometer. Cells were adjusted to 1 × 10^7^ cells/ml, and 3 μl of 10-fold serial dilutions was spotted on YPD agar plates supplemented with the compounds described in the figure legends. The plates were incubated at 37°C for 2 days and then photographed.

### Obtaining drug adaptors.

Cells of strain YJB-T12108 were adjusted to 1 × 10^7^ cells/ml as described above. Then, 100 μl of cell suspensions was spread on YPD plates supplemented with TUN (8 μg/ml) or AbA (0.1 μg/ml). The plates were incubated at 37°C for 3 days. Random colonies (*n* = 18) (adaptors) were chosen from each drug plate. The adaptors were streaked on YPD plates and grown at 37°C for 2 days. For each adaptor, several colonies with similar sizes were collected, preserved in 35% glycerol, and maintained in a −80°C freezer.

### Testing aneuploid stability.

Adaptors selected on plates containing drug were directly plated on YPD plates without drug. Most colonies were smaller than the parental strain. Large colonies appeared rarely, with a frequency of 0.03 to 0.4%. When adaptor isolates were re-streaked from frozen stocks, the frequency of large colonies was higher, ranging from 2% to 6%, but upon re-streaking of small colonies, the frequency returned to 0.03 to 0.4%, suggesting that the freeze/thaw process increased the aneuploid instability.

### Ploidy analysis.

Genomic DNA content was measured using flow cytometry as described in reference 42. Propidium iodide (PI) was used to stain the DNA. Cells were sonicated and analyzed in 96-well batches on a MACSQuant VYB instrument (Miltenyi Biotec). Data were analyzed using FlowJo software (version 10.4).

### Broth microdilution assay.

The broth microdilution assay was performed as described previously (44). For each strain, approximately 2.5 × 10^3^ cells/ml were grown in 150 μl YPD broth with or without the drugs in a 96-well plate at 37°C. The optical density at 595 nm (OD_595_) was monitored using a Tecan (Infinite F200 PRO; Tecan, Switzerland) plate reader at 15-min time intervals for 48 h.

### Next-generation sequencing.

The next-generation sequencing was performed as described previously (28). In order to visualize the karyotypes, raw fastq files were uploaded to YMAP version 1.0 (http://lovelace.cs.umn.edu/Ymap/), and the read depth was plotted as a function of chromosome location using the genome of C. parapsilosis strain CDC37 (version_s01-m03-r50) downloaded from http://www.candidagenome.org/download/sequence/C_parapsilosis_CDC317/current/ as the reference. Chromosome end bias and GC content bias were also corrected by YMAP as described in reference 41. In the karyotypes generated, Chr1, Chr2, Chr3, Chr4, Chr5, chr6, Chr7, and Chr8 correspond to Contig005504, Contig005569, Contig005806, Contig005807, Contig005809, Contig006110, Contig006139, and Contig006372, respectively.

### Data availability.

The genome sequence data are available at ArrayExpress at EMBL-EBI (www.ebi.ac.uk/arrayexpress) under accession numbers E-MTAB-10531 (TUN adaptors) and E-MTAB-10532 (AbA adaptors).
