# Supplementary material for: Aneuploidy Underlies Tolerance and Cross-Tolerance to Drugs in Candida parapsilosis
Source: Microbiol Spectr. 2021 Oct 6;9(2):e00508-21. doi: 10.1128/Spectrum.00508-21 (PMC8510177; doi:10.1128/Spectrum.00508-21)
Supplement: Supplemental file 1 — Supplemental material. Download SPECTRUM00508-21_Supp_1_seq1.pdf, PDF file, 1.9 MB [file spectrum00508-21_supp_1_seq1.pdf]

Supplemental materials

Figure S1. Karyotypes of tunicamycin adaptors

Karyotypes of the 18 tunicamycin adaptors (T1 – T18) were visualized using YMAP.

Figure S2. Tolerance of tunicamycin adaptors to tunicamycin and aureobasidin A.

Spot assay of tunicamycin adaptors tested on tunicamycin (TUN) and aureobasidin A (AbA). Plates were incubated at 37˚C for 2 days and then photographed.

Figure S3. Aureobasidin A adaptors are aneuploid and drug tolerant

Spot assay of 18 aureobasidin A (AbA) adaptors (A1 – A18) were tested on AbA and tunicamycin (TUN) (A). All were sequenced and the karyotypes were visualized using YMAP (B).

Figure S4. Broth microdilution assay

MICs of tunicamycin and aureobasidin A were measured for a Chr6x3 adaptor (small colony on YPD (S)), one large (L) colony from this adaptor, as well as the parent YJB-T12108. Broth microdilution assay was performed. Cells were grown in a 96-well plate in YPD broth supplemented with drugs as indicated. Optical density (OD_{595nm}) was measured using a Tecan plate reader (Infinite F200 PRO, Tecan, Switzerland) after 48h growth at 37˚C. The growth in wells with drug was normalized to growth in wells without drug.

Figure S5. Instability of tunicamycin and aureobasidin A adaptors

Adaptors representing each karyotype were streaked on YPD plates from glycerol stocks. The plates were incubated at 37˚C for 48h. Magenta arrows indicate the small colonies. Cyan arrows indicate the large colonies (A). For each adaptor, one small and one large colony were compared to the parent YJB-T12108 for tolerance to tunicamycin (TUN) or aureobasidin A (AbA) (B); the genomic DNA content for each of the large and

small colonies was measured by flow cytometry using propidium iodide (PI) staining of nuclear DNA (C).

Figure S1

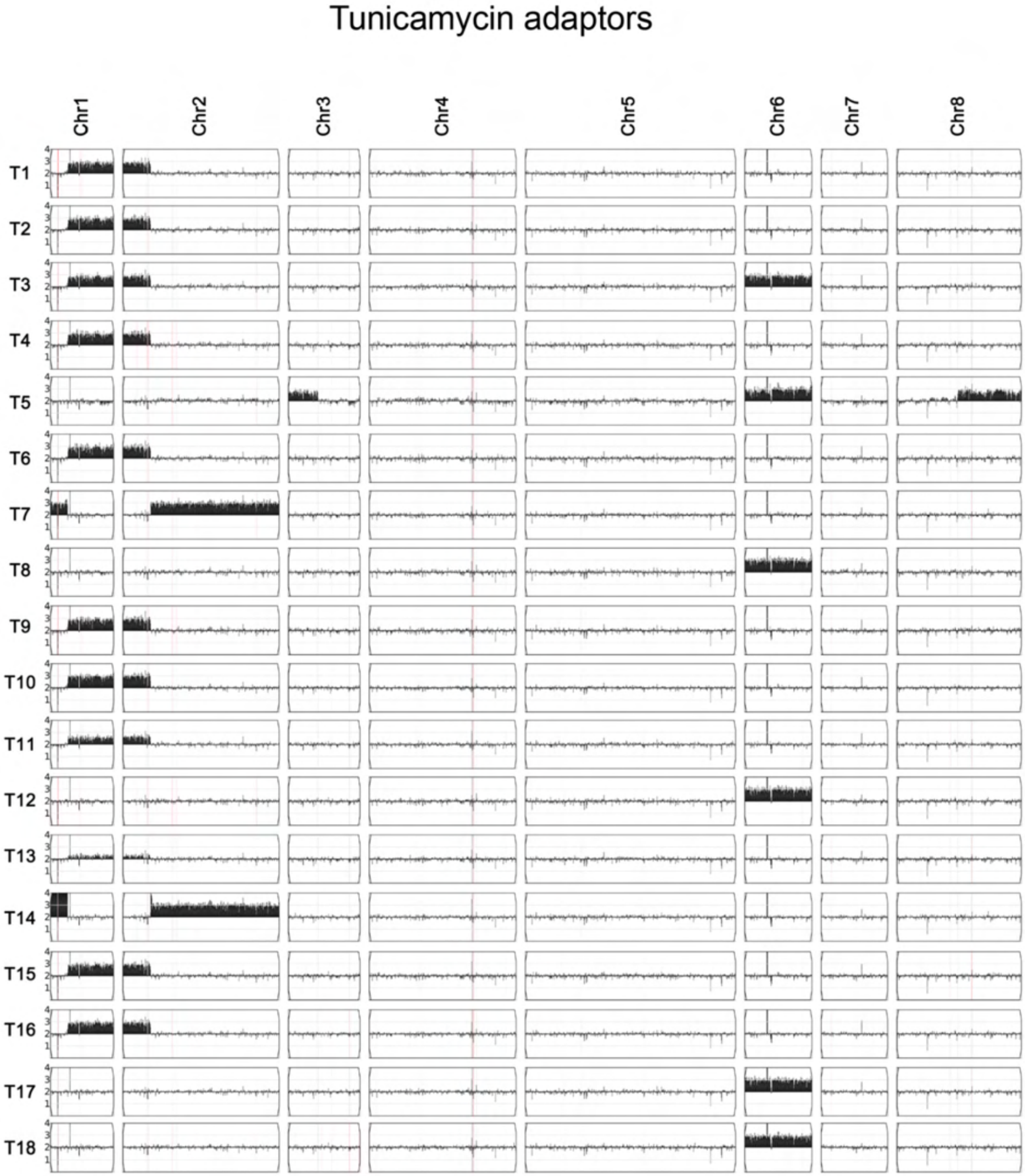
Tunicamycin adaptors
Chr1
Chr2
Chr3
Chr4
Chr5
Chr6
Chr7
Chr8
T1
T2
T3
T4
T5
T6
T7
T8
T9
T10
T11
T12
T13
T14
T15
T16
T17
T18

Figure S2

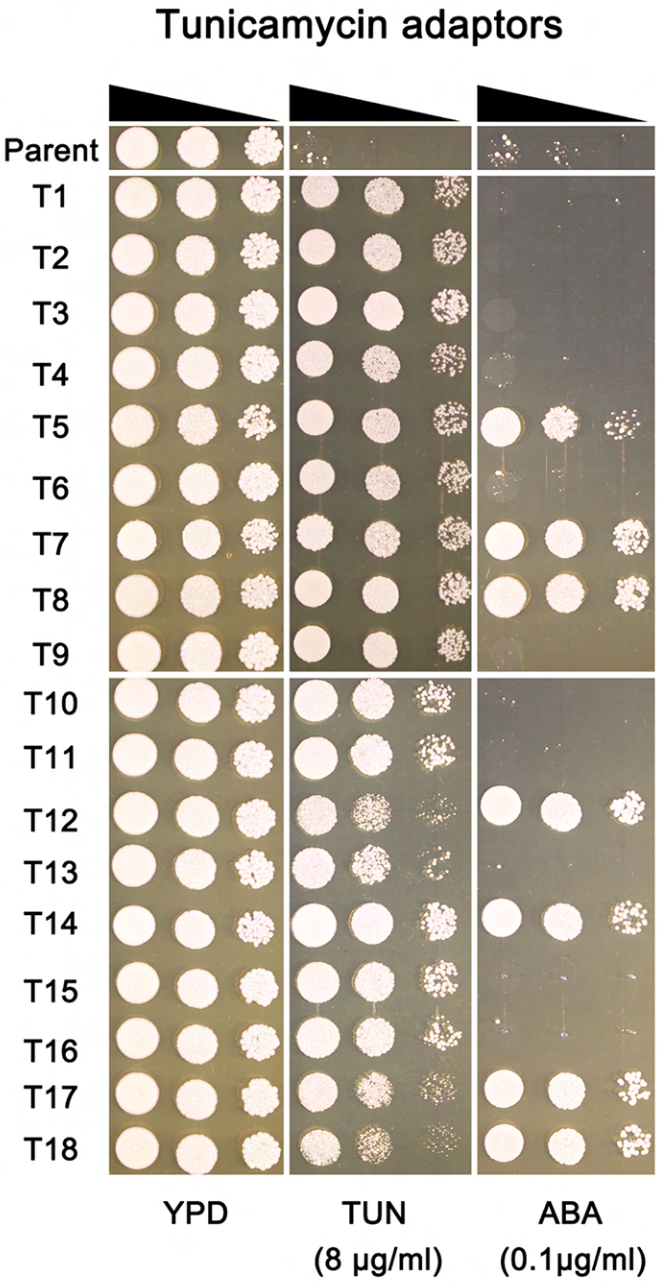
Tunicamycin adaptors
Parent
T1
T2
T3
T4
T5
T6
T7
T8
T9
T10
T11
T12
T13
T14
T15
T16
T17
T18
YPD
TUN
(8 µg/ml)
ABA
(0.1µg/ml)

Figure S3

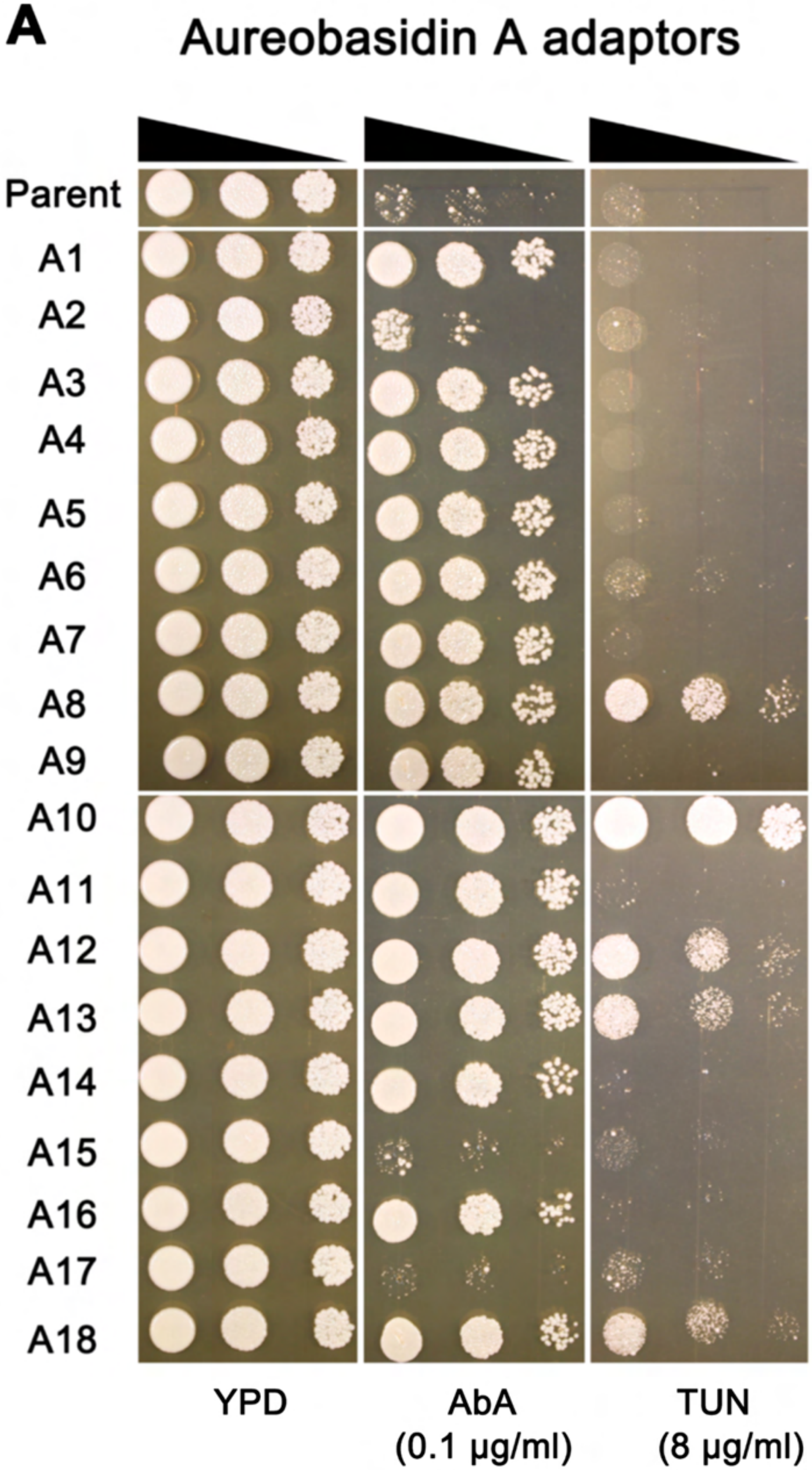
A
Aureobasidin A adaptors
Parent
A1
A2
A3
A4
A5
A6
A7
A8
A9
A10
A11
A12
A13
A14
A15
A16
A17
A18
YPD
AbA
(0.1 µg/ml)
TUN
(8 µg/ml)

B

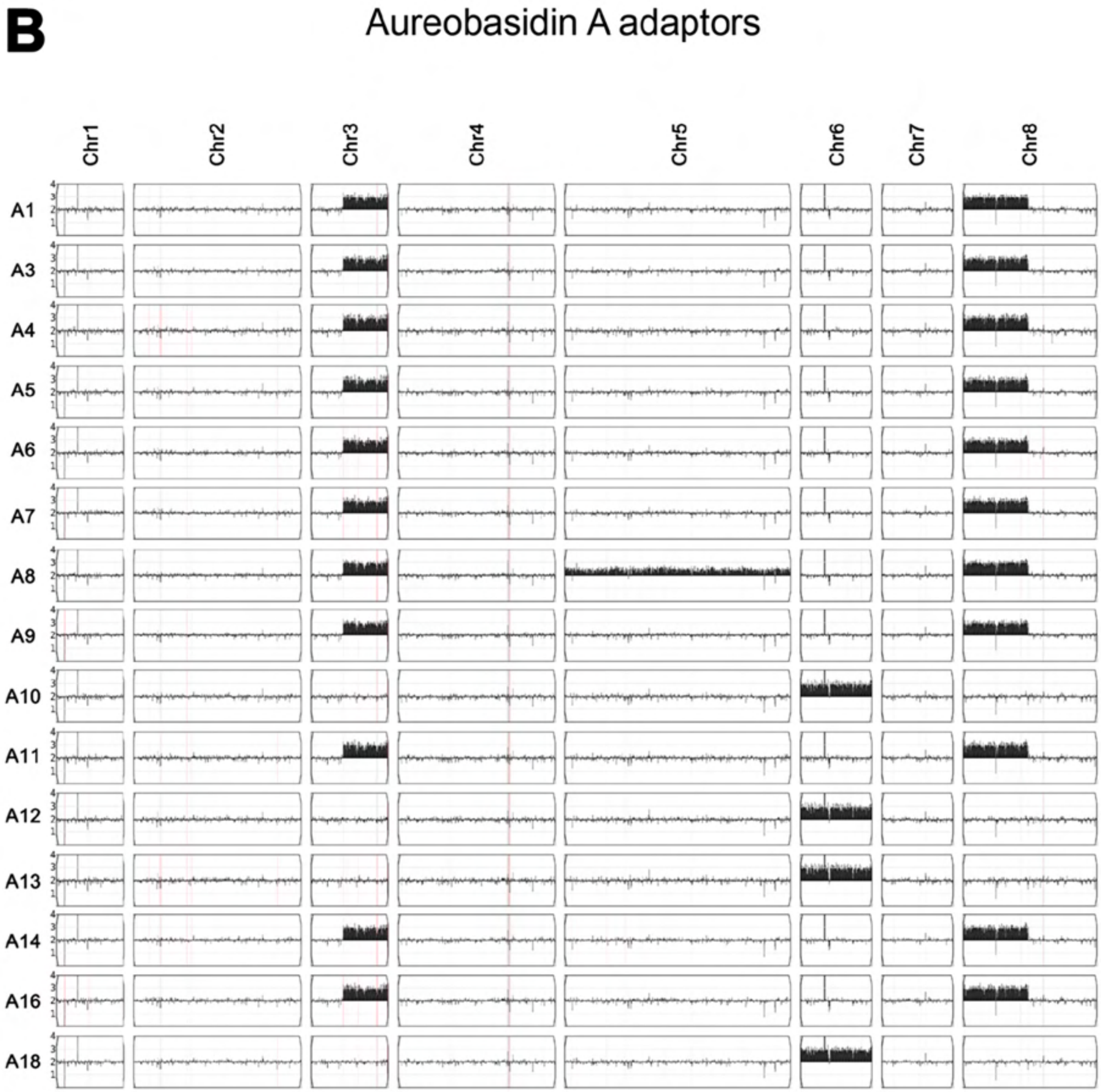
Aureobasidin A adaptors
Chr1
Chr2
Chr3
Chr4
Chr5
Chr6
Chr7
Chr8
A1
A3
A4
A5
A6
A7
A8
A9
A10
A11
A12
A13
A14
A16
A18

FiguFre S4

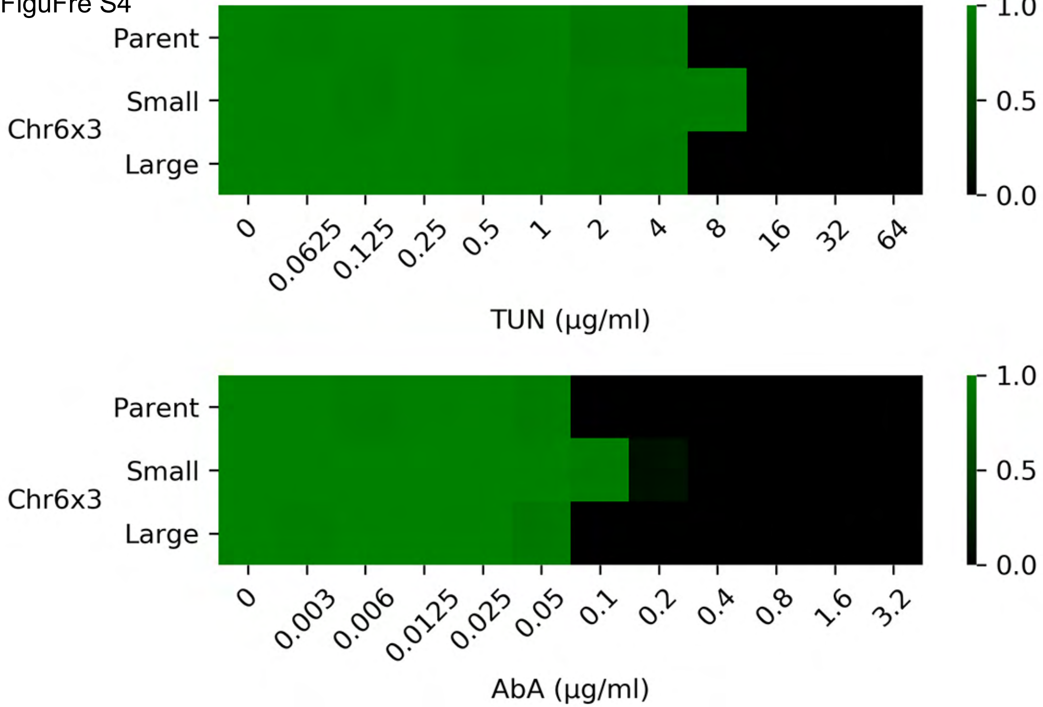
Parent
Small
Chr6x3
Large
0
0.0625
0.125
0.25
0.5
1
2
4
8
16
32
64
TUN (µg/ml)
1.0
0.5
0.0
Parent
Small
Chr6x3
Large
0
0.003
0.006
0.0125
0.025
0.05
0.1
0.2
0.4
0.8
1.6
3.2
AbA (µg/ml)
1.0
0.5
0.0

Figure S5

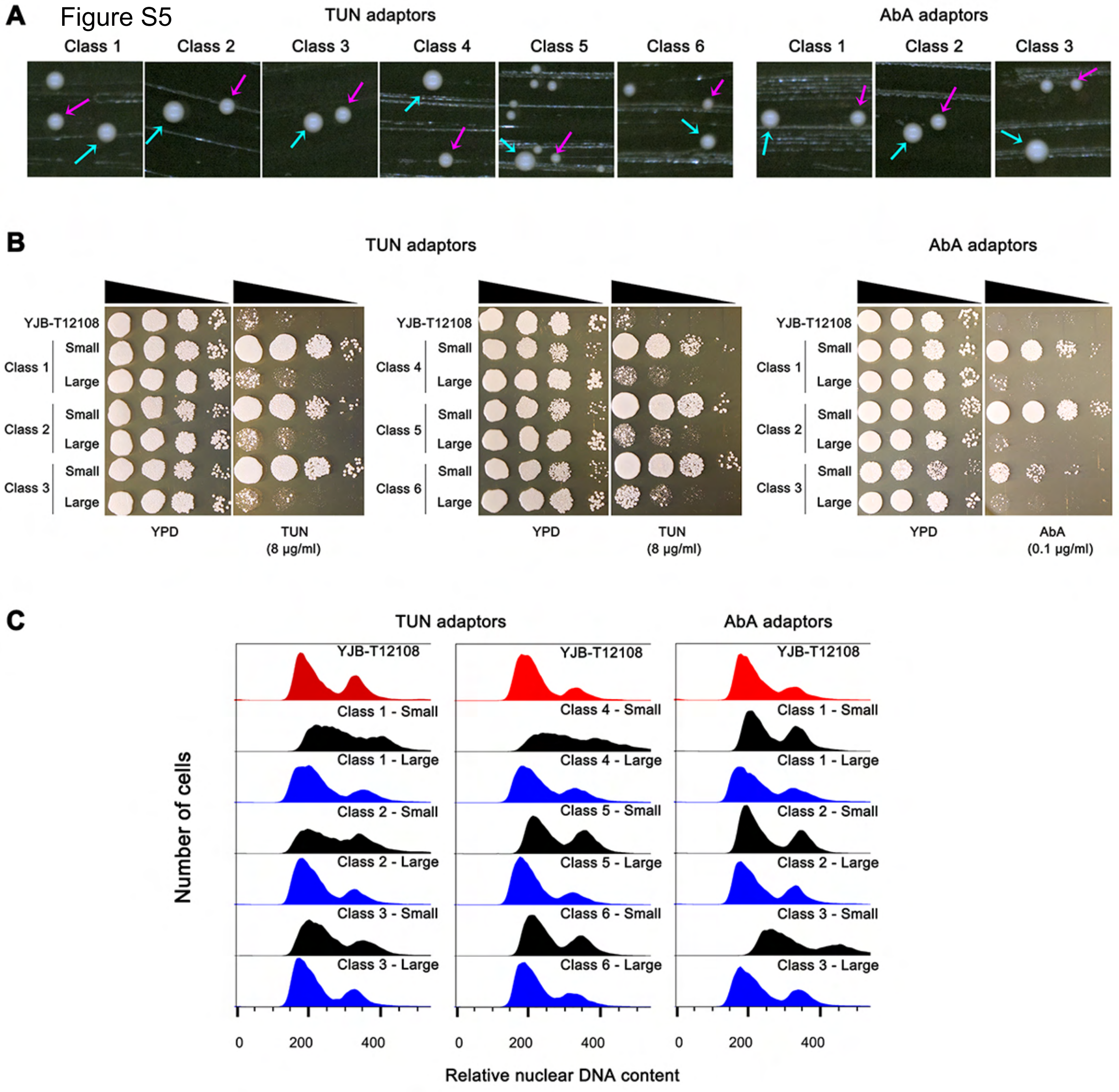
A
TUN adaptors
AbA adaptors
Class 1
Class 2
Class 3
Class 4
Class 5
Class 6
B
TUN adaptors
AbA adaptors
YJB-T12108
Small
Large
YPD
TUN
(8 µg/ml)
AbA
(0.1 µg/ml)
C
TUN adaptors
AbA adaptors
YJB-T12108
Class 1 - Small
Class 1 - Large
Class 2 - Small
Class 2 - Large
Class 3 - Small
Class 3 - Large
Class 4 - Small
Class 4 - Large
Class 5 - Small
Class 5 - Large
Class 6 - Small
Class 6 - Large
Number of cells
0
200
400
Relative nuclear DNA content
